# Gene expression profiles of liver cancer cell lines reveal two hepatocyte-like and fibroblast-like clusters

**DOI:** 10.1371/journal.pone.0245939

**Published:** 2021-02-04

**Authors:** Keita Fukuyama, Masataka Asagiri, Masahiro Sugimoto, Hiraki Tsushima, Satoru Seo, Kojiro Taura, Shinji Uemoto, Keiko Iwaisako

**Affiliations:** 1 Division of Hepato-Biliary-Pancreatic and Transplant Surgery, Department of Surgery, Graduate School of Medicine, Kyoto University, Kyoto, Japan; 2 Department of Pharmacology, Yamaguchi University Graduate School of Medicine, Ube, Japan; 3 Department of Experimental Immunology, Immunology Frontier Research Center, Osaka University, Suita, Japan; 4 Research and Development Center for Minimally Invasive Therapies Health Promotion and Preemptive Medicine, Tokyo Medical University, Shinjuku, Japan; 5 Department of Medical Life Systems, Faculty of Life and Medical Sciences, Doshisha University, Kyotanabe, Japan; University of Navarra School of Medicine and Center for Applied Medical Research (CIMA), SPAIN

## Abstract

Cancer cell lines are widely used in basic research to study cancer development, growth, invasion, or metastasis. They are also used for the development and screening of anticancer drugs. However, there are no clear criteria for choosing the most suitable cell lines among the wide variety of cancer cell lines commercially available for research, and the choice is often based on previously published reports. Here, we investigated the characteristics of liver cancer cell lines by analyzing the gene expression data available in the Cancer Cell Line Encyclopedia. Unsupervised clustering analysis of 28 liver cancer cell lines yielded two main clusters. One cluster showed a gene expression pattern similar to that of hepatocytes, and the other showed a pattern similar to that of fibroblasts. Analysis of hepatocellular carcinoma gene expression profiles available in The Cancer Genome Atlas showed that the gene expression patterns in most hepatoma tissues were similar to those in the hepatocyte-like cluster. With respect to liver cancer research, our findings may be useful for selecting an appropriate cell line for a specific study objective. Furthermore, our approach of utilizing a public database for comparing the properties of cell lines could be an attractive cell line selection strategy that can be applied to other fields of research.

## Introduction

Hepatocellular carcinoma (HCC) is the fifth most common cancer in the world, accounting for more than 700,000 new cases and 600,000 deaths globally per year [1]. Direct-acting antiviral agents with excellent efficacy are now available against hepatitis C virus, a major cause of HCC in the United States and Japan [2]. However, it is anticipated that HCC from nonalcoholic steatohepatitis will soon become the leading cause of HCC, with its incidence increasing in many countries [3]. Regardless of etiology, patients diagnosed with HCC at an advanced stage or with progression after locoregional therapy have a poor prognosis, owing to the lack of effective treatment options. There is, therefore, an urgent need for the development of effective therapies through efficient basic research.

One of the main issues with using cancer cell lines for research and development is their limited predictive value regarding the efficacy of novel treatments, and they are not always appropriate as cancer models [4]. In preclinical study-based development using cell lines, the average response rate of a phase I clinical trial has been reported to be 3%, even if cell proliferation is completely suppressed in vitro [5,6]. Only 13.4% of anticancer agents have been proven to be effective upon progression to phase III clinical trials [7]. Sorafenib was the first drug recommended in the clinical practice guidelines developed by the European Association for the Study of the Liver, the European Organization for Research and Treatment of Cancer [8], and the American Association for the Study of Liver Diseases [9]. In preclinical in vitro studies, sorafenib has been reported to affect the viability of HCC cell lines. Sorafenib-treated PLC/PRF/5 cells demonstrated apoptotic change, with nuclear condensation and fragmentation, which was not observed in sorafenib-treated HepG2 cells [10]. Moreover, treatment with sphingosine kinase 2 inhibitor and sorafenib induced higher activation of caspases 3/7 in HepG2 cells than in Hep3B2.1–7 cells, indicating that the apoptotic response to these compounds varies among HCC cells [11]. As different effects were observed depending on the cell line used, the choice of cell lines is considered an important factor in experiments assessing drug efficacy.

Cancer cell lines are established from cancer tissues. In preclinical studies, the method for choosing a suitable cell line for the study is unclear, aside from selection based on the organ the cell line originated from. We suspected that some cancer cell lines have biological characteristics that differ from those of cancer cells from the original tumor tissue. To investigate this hypothesis, we used gene expression data available in the Cancer Cell Line Encyclopedia (CCLE) and The Cancer Genome Atlas (TCGA), both of which are public databases. The CCLE includes the genomic and gene expression profiles of 947 cell lines [12]. TCGA Research Network has characterized the gene expression profiles of over 10,000 human tissue samples across 32 tumor types (https://www.cancer.gov/about-nci/organization/ccg/research/structural-genomics/tcga). Given the benefits of current technologies, we were able to take full advantage of these detailed datasets. To determine unknown biological characteristics, unsupervised clustering based on transcriptome similarity has emerged as a powerful application of these datasets. In some cases, cancer cell lines are used as a surrogate for epithelial cells. Moreover, the dataset of the atlas of human primary cells can be used for comparing gene expression profiles between cancerous and non-cancerous cells [12].

In this study, we compared the gene expression profiles of HCC from CCLE and TCGA and identified the differential gene expression (DGE) patterns. Based on our findings, a suitable cell line can be selected for in vitro HCC studies. Although conclusions based on in vitro cell line experiments are not necessarily valid in a clinical setting, choosing cell lines that are most representative of HCC should increase the value of cell line studies in preclinical research. Cell lines that were used to verify molecularly targeted drugs that were later approved as therapeutic agents for HCC were classified as hepatocyte-like in this study Our findings should indicate the appropriateness of hepatocyte-like cell lines for developing effective treatments for HCC. This study presents a useful method that can be utilized for drug-development research using cell lines.

## Materials and methods

### Data acquisition

mRNA expression data (CCLE_Expression.Arrays_2013-03-18.tar.gz) and annotation files (CCLE_Expression.Arrays.sif_2012-10-18.txt) were downloaded from the CCLE (https://portals.broadinstitute.org/ccle). The Catalogue of Somatic Mutations in Cancer (COSMIC) data were downloaded from the ENBL-EBI website (https://www.ebi.ac.uk/arrayexpress/files/E-MTAB-3610/). The microarray data were normalized using the MAS5 or RMA method, and log2 conversion was performed for the signal intensity of each probe set ID. RNA-seq data and annotation data were downloaded from the NCI website (ftp://caftpd.nci.nih.gov/pub/OCG-DCC/CTD2/TGen/CCLE_RNA-seq_Analysis/).

RNA-seq data and clinical information regarding HCC from TCGA, including the normalized transcripts per million (TPM) data for clustering and visualization (http://gdac.broadinstitute.org/runs/stddata__2016_01_28/data/LIHC/20160128/gdac.broadinstitute.org_LIHC.Merge_rnaseqv2__illuminahiseq_rnaseqv2__unc_edu__Level_3__RSEM_genes_normalized__data.Level_3.2016012800.0.0.tar.gz), raw count data for identification of DEGs (http://gdac.broadinstitute.org/runs/stddata__2016_01_28/data/LIHC/20160128/gdac.broadinstitute.org_LIHC.Merge_rnaseqv2__illuminahiseq_rnaseqv2__unc_edu__Level_3__RSEM_genes__data.Level_3.2016012800.0.0.tar.gz), and clinical information for annotation (http://gdac.broadinstitute.org/runs/stddata__2016_01_28/data/LIHC/20160128/gdac.broadinstitute.org_LIHC.Clinical_Pick_Tier1.Level_4.2016012800.0.0.tar.gz) were obtained from the Broad Institute website (https://gdac.broadinstitute.org/).

Clinical TCGA information was obtained from the TCGA legacy archive (https://portal.gdc.cancer.gov/legacy-archive/search/f, S1 Table). Gene expression data of various primary cells were downloaded from the National Center for Biotechnology Information Gene Expression Omnibus (https://www.ncbi.nlm.nih.gov/gds/?term=GSE49910 [Accession]).

### Clustering

We performed unsupervised hierarchical clustering analysis for mRNA expression data of cancer cell lines, HCC samples, or primary cells using Spearman correlation and average linkage or Ward’s method. We uploaded the code used in this study to GitHub (https://github.com/fk506cni/cell_line_comparison/tree/master/analysis/77GEarray_re).

### Literature review of liver cancer cell lines

Original articles on the establishment of liver cancer cell lines were searched [13–25], and the age of the patients was determined using the Brunner-Munzel test.

### Identification of differentially expressed genes (DEGs)

We examined the difference in the expression data of the two liver-cancer cell-line clusters. The corrected p-values (obtained by moderated t-statistics using the eBayes function of the Bioconductor package “limma”) and the q-values (adjusted by the Benjamini-Hochberg method) were calculated for each probe set ID. DEGs were defined as probe set IDs with q-values <0.0001. Additionally, we classified DEGs by the difference in the log2 average between the two clusters. DEGs with higher expression of cluster A cell lines than those of cluster B were defined as A genes, and those with higher expression of cluster B cell lines were defined as B genes.

### Principal component analysis (PCA)

Principal component analysis (PCA) for DEGs was performed to confirm the ability of our approach to distinguish the cell types in clusters A and B. Further, we carried out a discriminant analysis of the principal components and evaluated the validity of the clusters using the *adegenet* package in R.

### Comparison of DEGs from RNA-seq data

We evaluated the DEGs in the HCC RNA-seq data acquired from TCGA. For visualization, we used log-transformed TPM as the expression level [specifically log2(TPM + 1)]. For clinical samples from TCGA, the fold change between the two clusters was calculated. Genes were then sorted according to fold change after subtracting the average expression level between two clusters.

### TCGA clinical data analysis

We analyzed the clinical data of patients classified into two clusters defined based on cell line analysis. The weight of the surgical specimen, a continuous variable, was assessed using the Brunner-Munzel test. The operating procedure, a categorical variable, was assessed using Fisher’s exact test. Cox proportional-hazards model and log-rank test were used for survival analysis. A p-value of less than 0.05 was considered statistically significant.

### Gene Ontology (GO) enrichment analysis

We performed GO enrichment analysis of the A and B genes through DAVID (https://david.ncifcrf.gov/) via the Bioconductor “RDAVIDWebService” package, with a Benjamini-Hochberg-adjusted p-value < 0.2. We used “GOTERM_BP_FAT” level annotation. The top ten GO terms were visualized using bar plots, with the q-value and the number of enriched genes.

### Mapping to primary cell atlas

We normalized all CEL files, including liver cancer cell lines and primary cells, and integrated them. Sample categories were annotated from the metadata. Expression levels were visualized using a heatmap.

### Calculation of representative vectors for each cell type

The vector of the expression pattern for each cell type was generated as the log2 median of each probe set ID value (S1 Document).

### Statistical analysis

Statistical analysis and data visualization of the expression array and RNA-seq were performed on the R studio (version 1.2.5042) server and R studio (1.3.959) (https://rstudio.com/) using several R (version 3.4.1, 3.5.3, and 3.6.3) and Bioconductor (3.5 and 3.6) packages (S1 Document). The code was deposited in GitHub (https://github.com/fk506cni/cell_line_comparison). Our protocols were deposited in protocols.io (dx.doi.org/10.17504/protocols.io.bq5vmy66). Comparison of a continuous variable in 2 groups was assessed using the Brunner-Munzel test. Qualitative data were compared using the Fisher’s exact test to compare categorical variables. A p-value of less than 0.05 was considered statistically significant.

## Results

### Clustering of gene expression patterns in digestive cancer cell lines

**Fig 1:**
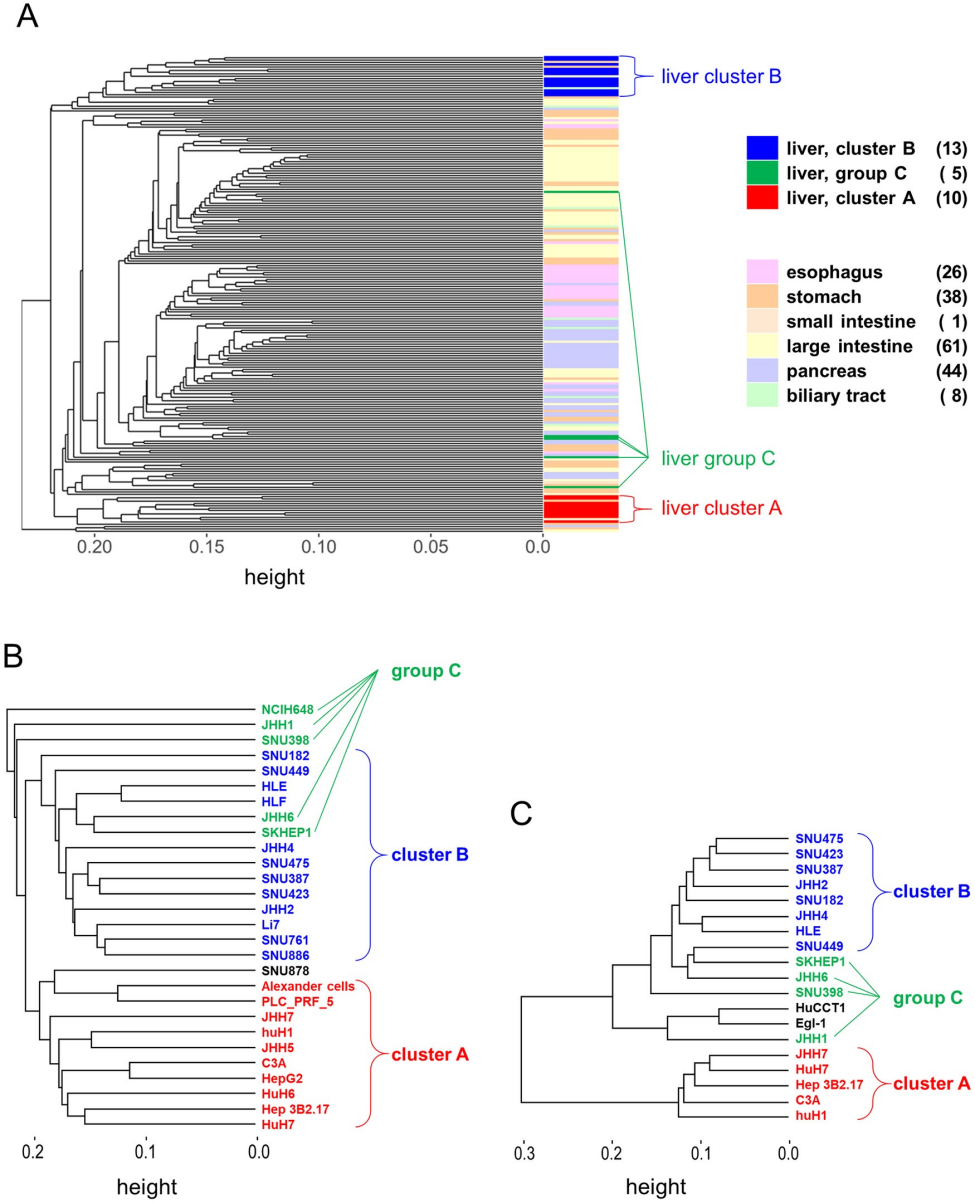
Expression-based unsupervised hierarchical clustering. (A) The expression data of 206 digestive cancer cell lines from the Cancer Cell Line Encyclopedia (CCLE) were analyzed. (B) The 28 liver cancer cell lines obtained from CCLE were extracted and analyzed again. (C) Nineteen liver cancer cell lines in another dataset from the Catalogue of Somatic Mutations in Cancer (COSMIC) were clustered.

We performed unsupervised clustering analysis using the expression data of 206 digestive cancer cell lines, which were profiled using microarray, the data for which were deposited in the CCLE. The results of clustering were compared with those for the original organs from which the cell lines were derived (Fig 1A and S2 Table). Among all digestive cancer cell lines, liver cancer cell lines showed two major clusters, designated as A and B for convenience. Ten liver cancer cell lines were classified under cluster A, and 13 were classified under cluster B. The other five were identified as cell lines derived from other organs and were classified as group C. Cluster A included HepG2, the most widely used liver cancer cell line. After extracting the 28 liver cancer cell lines from 206 digestive cancer cell lines, we performed clustering analysis again (Fig 1B). Similar to the previous analysis, the same major two clusters were formed. These findings suggest that there are at least two expression patterns in liver cancer cell lines. Moreover, we observed two liver cancer cell line clusters using different datasets from the COSMIC (Fig 1C).

### Literature review of the origin of liver cancer cell lines

We reviewed reports concerning the establishment of liver cancer cell lines (Table 1). Cell lines in cluster A originated from patients who were younger than those for cluster B (p < 0.05). Cluster A contains three cell lines (HuH6, HepG2, and C3A) established from HCC patients no older than 15 years. The nine Seoul National University (SNU) cell lines were established from Asian HCC patients infected with the hepatitis B virus, and eight of these were classified under cluster B.

**Table 1:** Details of liver cancer cell lines.

Cell line	Gender	Ethnicity	Age	Primary tumor	Cluster	Viral status	Preoperative treatment	Reference	Year of publication
**Alexander cells**	M	Black	24	Hepatoma	A	HBV+	no	13	1976
**C3A**	M	Caucasian	15	Differentiated HCC	A	non	no	14	1979
**Hep 3B2.1–7**	M	Black	8	Differentiated HCC	A	HBV+	no	14	1979
**Hep G2**	M	Black	15	Differentiated HCC	A	non	no	14	1979
**huH1**	M	Asian	53	Hepatoma	A	HBV+	no	15	1981
**HuH6**	M	Asian	Infant	Hepatoblastoma	A	non	no	16	1976
**HuH7**	M	Asian	57	Well-differentiated HCC	A	non	no	17	1982
**JHH5**	M	Asian	50	HCC	A	non	no	18	1990
**JHH7**	M	Asian	53	HCC	A	HBV+	no	18	1990
**PLC_PRF_5**	M	Black	24	Hepatoma	A	HBV+	no	13	1976
**HLE**	M	Asian	68	Undifferentiated HCC	B	non	no	19	1975
**HLF**	M	Asian	68	Undifferentiated HCC	B	non	no	19	1975
**JHH2**	M	Asian	57	HCC	B	non	no	18	1990
**JHH4**	M	Asian	51	HCC	B	non	no	18	1990
**Li7**	M	Asian	45	Thick trabecular HCC	B	non	no	20	1979
**SNU182**	M	Asian	24	HCC, grade III/IV	B	HBV+	no	21	1995
**SNU387**	F	Asian	41	HCC, grade III-IV/IV	B	HBV+	TACE	21	1995
**SNU423**	M	Asian	40	HCC, grade III/IV	B	HBV+	TACE	21	1995
**SNU449**	M	Asian	52	HCC, grade II-III/IV	B	HBV+	no	21	1995
**SNU475**	M	Asian	43	HCC, grade III-IV/IV	B	HBV+	no	21	1995
**SNU761**	M	Asian	49	HCC, grade III/IV	B	HBV+	TAE	22	1999
**SNU878**	F	Asian	54	HCC, grade II III/IV	B	HBV+	TAE	22	1999
**SNU886**	M	Asian	57	HCC, grade II IV/IV	B	HBV+	TAE	22	1999
**JHH1**	M	Asian	50	HCC, Ed. III	C	non	no	23	1985
**JHH6**	F	Asian	57	HCC, Ed. II	C	non	no	18	1990
**NCIH648**	M	Caucasian	55	Adenocarcinoma*	C	non	no	24	1987
**SKHEP1**	M	Caucasian	52	Adenocarcinoma	C	non	NA	25	1977
**SNU398**	M	Asian	42	HCC	C	HBV+	TACE	21	1995

*Although NCIH648 is classified as liver cancer in CCLE, it was established from the liver metastatic tumor of colon cancer.

Abbreviations: HBV, hepatitis B virus; HCC, hepatocellular carcinoma.

TAE; transarterial embolization, TACE; transarterial chemoembolization.

### DEGs in the two clusters

We identified DEGs between the two liver cancer cell line clusters A and B. For this analysis, SNU878 was excluded owing to its inconsistent results. After clustering all 206 cell lines, SNU878 was classified under cluster B. However, after clustering the 28 liver cancer cell lines, SNU878 was classified under cluster A. SUN-878 had an intermediate profile between the two clusters. Furthermore, five group C cell lines were also omitted. Thus, the gene expression data of 22 liver cancer cell lines were provided for the analysis.

**Fig 2:**
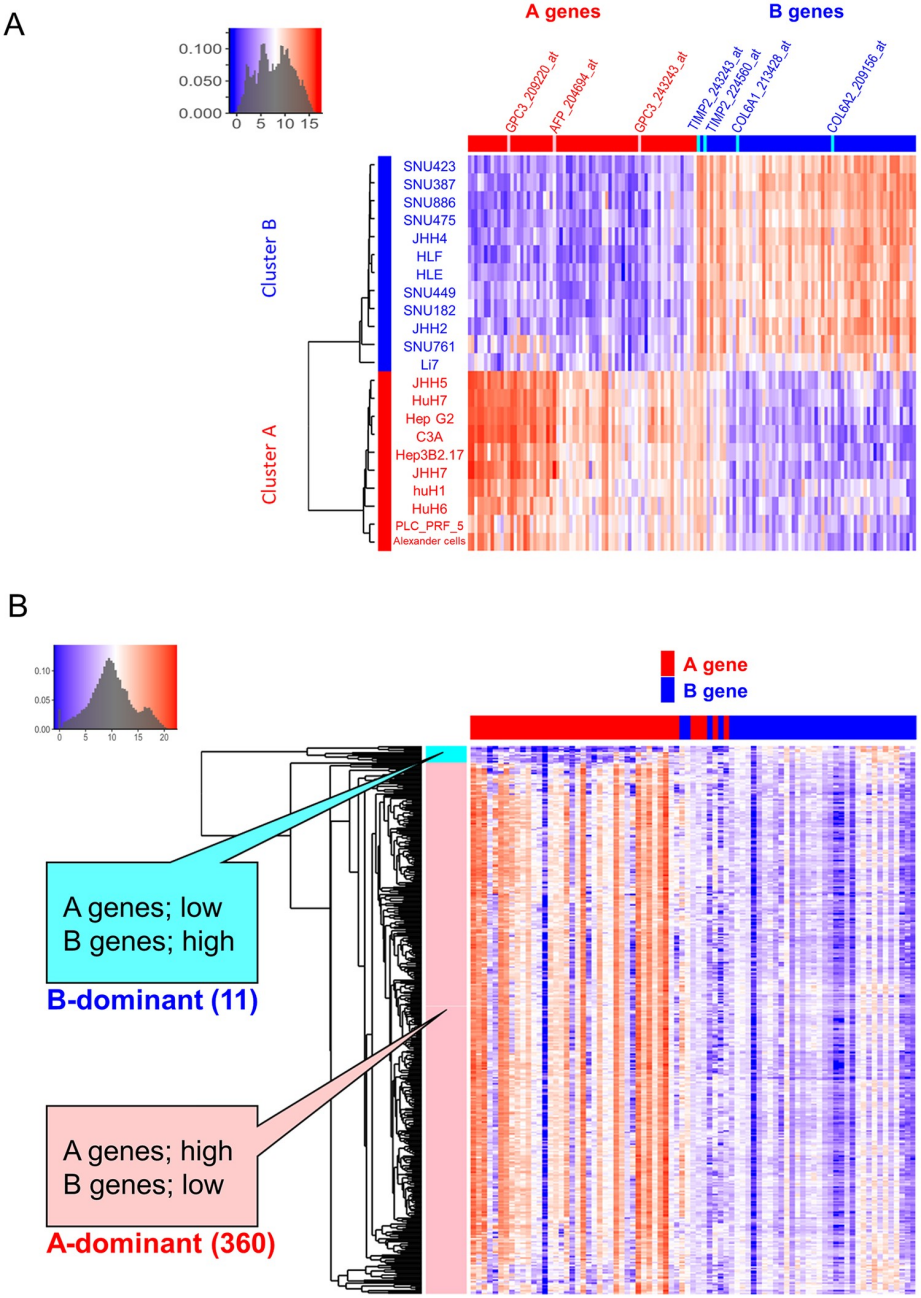
Heatmap visualization and hierarchical clustering of the selected differentially expressed genes. Heatmap showing the hierarchical clustering of (A) 22 liver cancer cell lines from the Cancer Cell Line Encyclopedia (CCLE) and (B) 371 hepatocellular carcinoma (HCC) tumor samples from the Cancer Genome Atlas (TCGA) based on their gene expression. Each row represents one sample (cell line or HCC), and each column represents the expression level of one gene.

We defined genes showing a q-value <0.0001 as a DEG. DEGs were classified by the difference in the average expression value in the two clusters. DEGs that were expressed more highly in cluster A than in cluster B were classified as A genes, and their counterparts were classified as B genes. Seventy probe set IDs containing 43 unique gene symbols were classified as A genes, and 38 gene symbols consisting of 67 probe set IDs were classified as B genes (Fig 2A and S3 Table). AFP and GPC3, which are known tumor markers for HCC, were classified as A genes. The B genes contained COL6A1 and COL6A2, which encode procollagen type VI and TIMP2 encoding matrix metalloprotease inhibitor molecules.

We performed PCA to examine the ability of our approach to distinguish the cell types in clusters A and B (S1 Fig). The PCA score plots obtained using the first two components were consistent with the clustering data and separated the liver cancer cell lines into two clusters: cluster A and cluster B. Furthermore, discriminant analysis of the principal components was used to validate our approach and resulted that the optimal number of clusters for this dataset was two.

### Comparison of gene expression patterns between liver cancer cell lines and hepatoma tissues

We used RNA-seq data of HCC deposited in TCGA to analyze the DEGs obtained from the liver cancer cell line analysis (Fig 2B and S4 Table). Unsupervised clustering of the 371 HCC samples resulted in two clusters. One cluster showed high A gene expression and low B gene expression, which was classified as A-dominant. The opposite pattern was observed for the other cluster, which was denoted B-dominant. However, the proportion of HCC samples in the two clusters was not the same as that observed for the liver cancer cell lines. Almost all samples showed an A-dominant pattern, and only 11/371 samples showed the opposite B-dominant pattern.

We collected the clinical information of HCC from TCGA. Half of the patients in the A-dominant group had a tumor pathologically diagnosed as T1 (solitary tumor ≤ 2 cm or > 2 cm without vascular invasion), whereas the B-dominant group did not include T1 (Table 2).

**Table 2:** Clinical information of HCC of TCGA.

Variable	Category	A dominant	B dominant	p-value
**pT**	T1	181	0	0.003
	T2	89	5	
	T3	75	5	
	T4	12	1	
	NA	3	0	
**Stage**	I	179	0	0.003
	II	88	4	
	III	85	7	
	IV	5	0	
	NA	3	0	
**Operative procedure**	extended_lobectomy	22	3	0.018
	lobectomy	137	5	
	other	24	2	
	segmentectomy_multiple	86	1	
	segmentectomy_single	87	0	
	total.hepatectomy.with.transplant	1	0	
	NA	3	0	

### GO enrichment and comparison with primary cells

**Fig 3:**
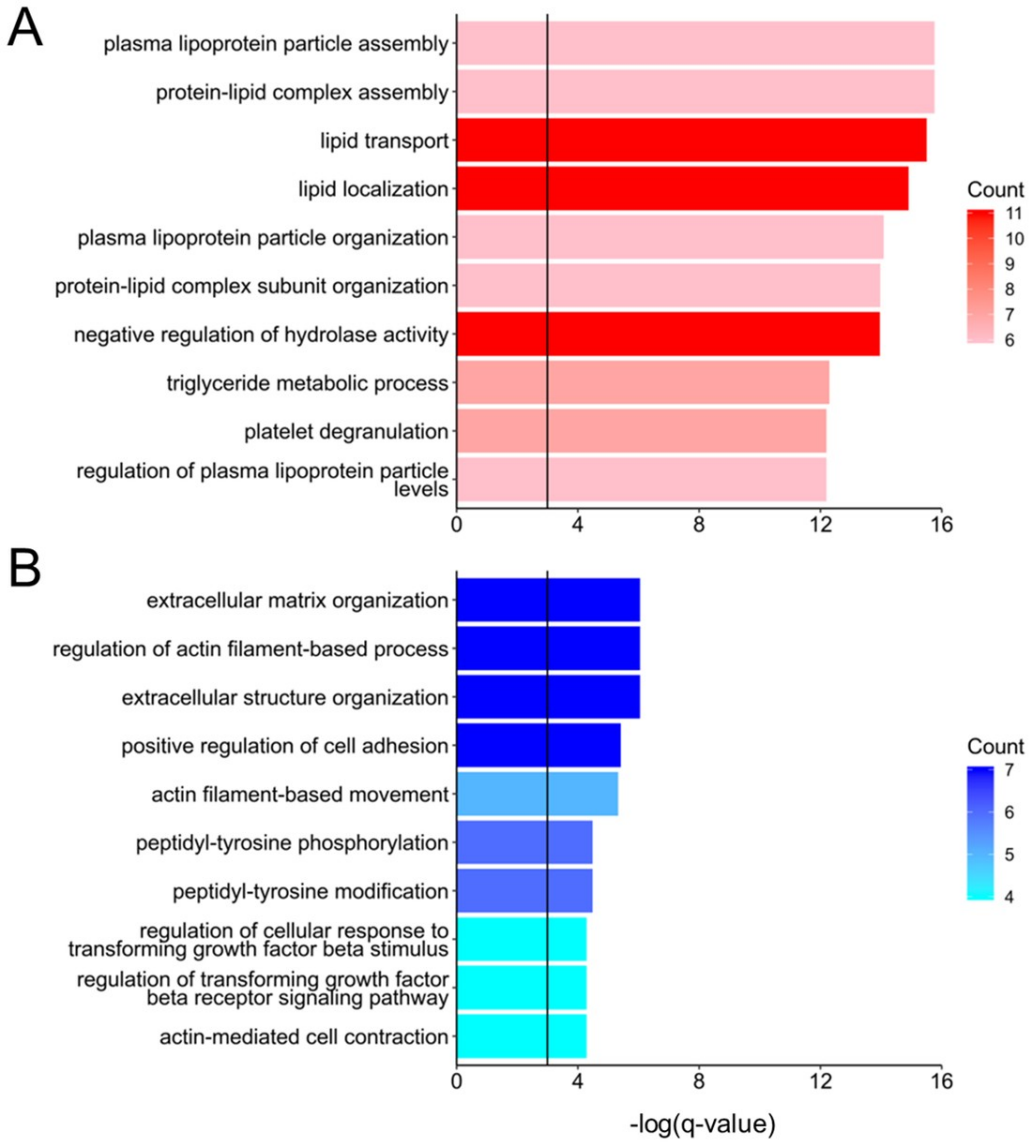
Gene Ontology (GO) analysis of A and B genes using DAVID. The enriched terms in (A) the A genes are indicated by a reddish column, and those in (B) the B genes are indicated by a bluish column. The color depth corresponds to the number of counts. Black vertical lines show the -log(0.05) results for (A) A genes and (B) B genes.

We performed GO enrichment analysis for the A and B genes using DAVID. Enriched terms in the A genes were associated with lipid metabolism, and those in the B genes were associated with extracellular matrix organization and cell adhesion (Fig 3). Although lipid metabolism is known to be a function of hepatocytes, non-parenchymal cells are responsible for extracellular matrix organization.

**Fig 4:**
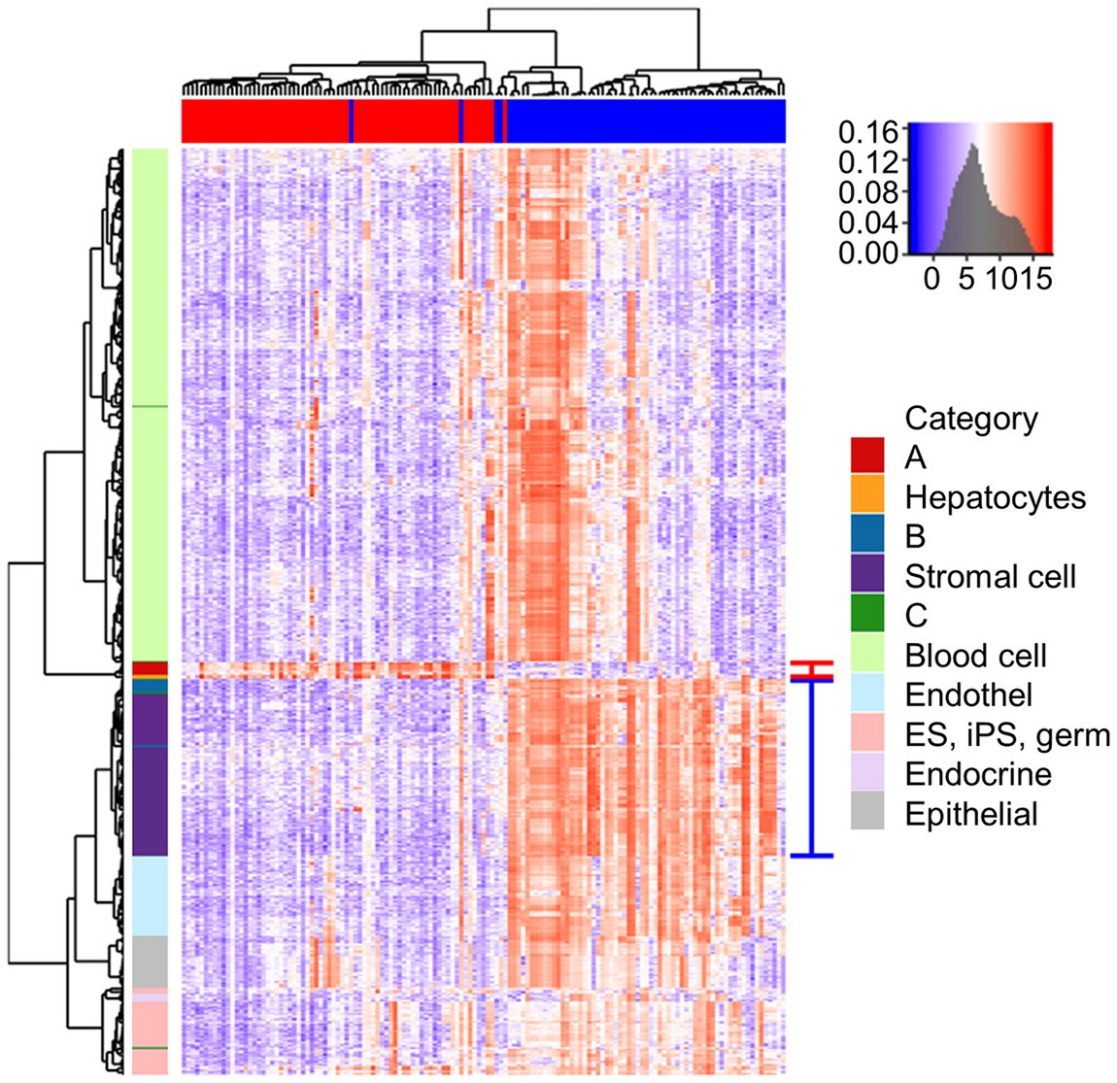
Unsupervised hierarchical clustering of liver cancer cell lines and primary cells. The cluster, including cell line A and hepatocytes, is indicated with a red line on the right, and the cluster, including cell line B and stromal cells, is indicated with a blue line.

We integrated the expression data of liver cancer cell lines and primary cells to analyze the DEGs that allowed us to investigate similar cellular properties. We used datasets from the CCLE and the atlas of human primary cells [26]. Cell lines in cluster A and primary hepatocytes appeared next to each other and showed a similar gene expression pattern. Cell lines in cluster B and stromal cells had similar gene expression patterns. The cell lines were then grouped and connected by a series of branches (Fig 4 and S5 Table).

**Fig 5:**
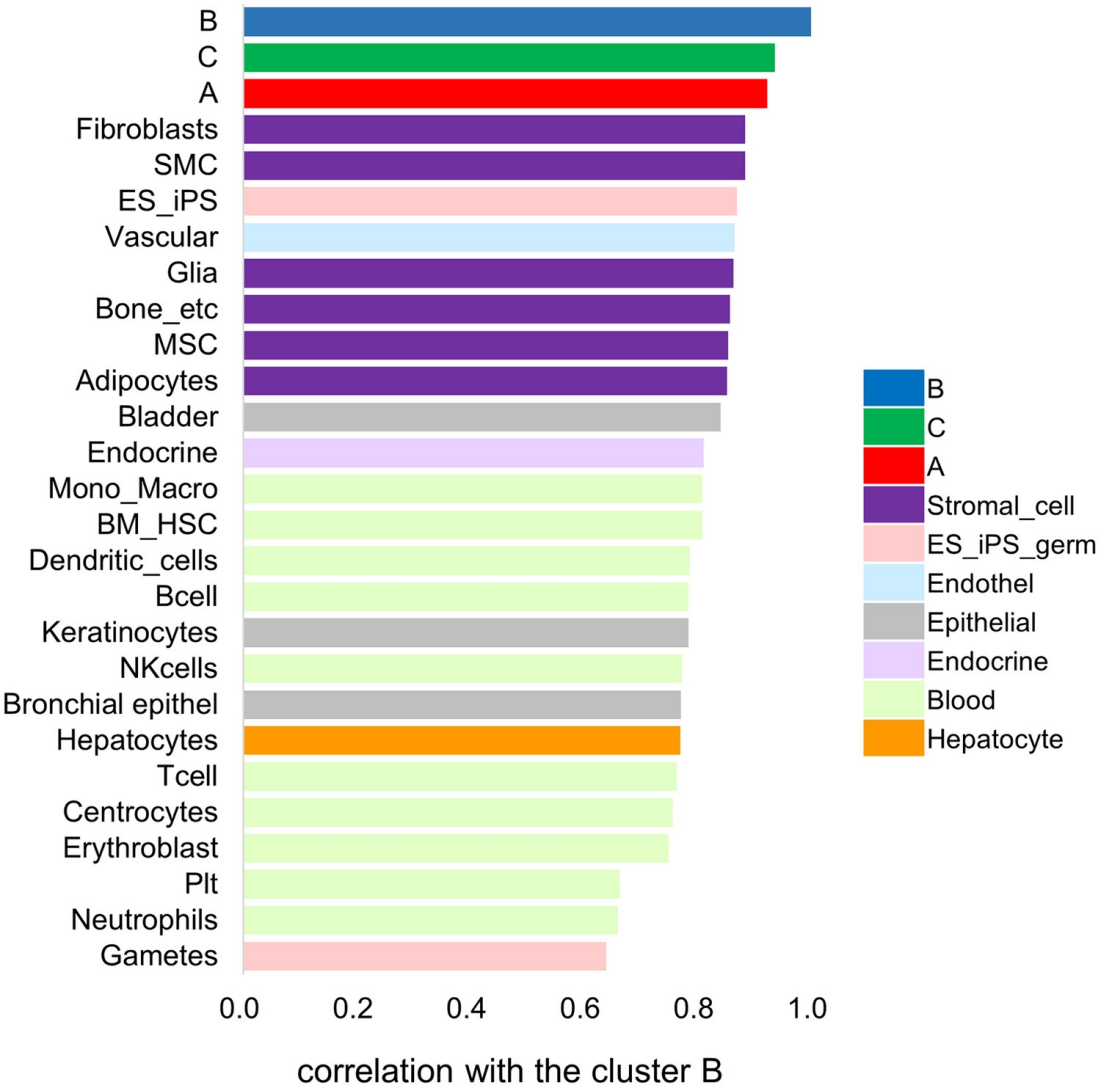
Comparison of the vector values of the expression pattern for each cell type. The graph of vector values shows primary cells that resemble cluster B cell lines. The color of columns for each cell corresponds to the cell category, same as in Fig 4.

We constructed a representative vector from several classes of primary cells and clusters of cell lines to investigate which cells categorized as stromal cells resemble the cluster B gene expression pattern. This vector is presented as the log2 median signal intensity of the expression values of each probe set ID. The correlation analysis was performed among the representative vectors of cells, and primary fibroblasts were found to be similar to the cluster B cell lines. However, in this analysis, group C was the most similar to cluster B, followed by cluster A. Therefore, high similarity was confirmed among the hepatoma cell lines (Fig 5).

The results of GO enrichment analysis, DEG analysis, and correlation analysis revealed that the gene expression pattern of cluster A is similar to that of hepatocytes, and the gene expression pattern of cluster B is similar to that of fibroblasts.

### Fibroblast-like cancer cell lines in the CCLE

**Fig 6:**
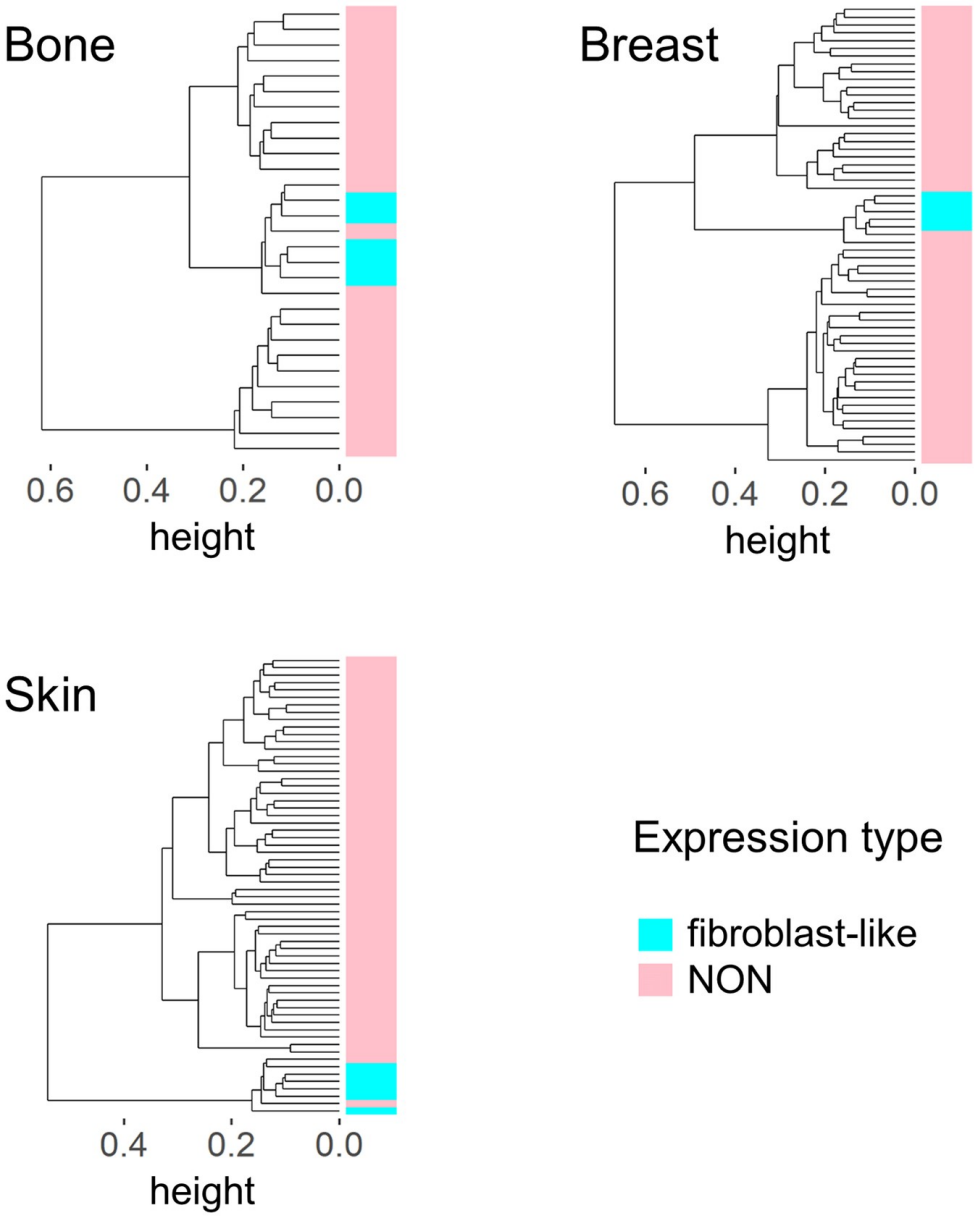
Unsupervised clustering analysis of cell lines established from bone, breast, or skin cancer. A cluster of fibroblast-like cell lines was observed regardless of the origin. In correlation analysis with the representative vector, setting the value of primary fibroblasts to 1, a fibroblast-like cell line has a value >0.88. Those with a value of 0.88 or less were classified as NON.

We analyzed 947 cancer cell lines in the CCLE to identify fibroblast-like cell lines by correlation analysis with the representative vector. Setting the value of primary fibroblasts to 1, only 23 cell lines with values >0.88 in cluster B were identified (Table 3). From the viewpoint of origin, bone, skin, and breast contain five or more fibroblast-like cell lines. Unsupervised clustering by each origin showed that fibroblast-like cell lines determined by correlation analysis formed one cluster (Fig 6 and S6 Table), suggesting the presence of a cluster of cancer cell lines with a fibroblast-like gene expression pattern, not only in the liver but also in other organs.

**Table 3:** Correlation with the representative vector of fibroblasts.

Cell line	Origin	Correlation with the representative fibroblast vector	Disease
**Primary fibroblast**		1.00	
**HS834T**	Skin	0.90	Melanoma
**HS606T**	Breast	0.90	Carcinoma
**HS281T**	Breast	0.90	Adenocarcinoma
**HS688AT**	Skin	0.90	Melanoma
**RS5**	Pleura	0.90	Pleural sarcomatoid mesothelioma
**HS600T**	Skin	0.90	Malignant melanoma
**HS934T**	Skin	0.90	Malignant melanoma
**HS274T**	Breast	0.90	Adenocarcinoma
**DM3**	Pleura	0.89	Mesothelioma
**HS343T**	Breast	0.89	Adenocarcinoma
**HS698T**	Large_Intestine	0.89	Adenocarcinoma
**HS616T**	Hematopoietic and lymphoid tissue	0.89	Hodgkin’s lymphoma
**HS863T**	Bone	0.89	Ewing’s sarcoma
**HS888T**	Bone	0.89	Osteo sarcoma
**BJHTERT**	Skin	0.89	(Foreskin fibroblasts transfected hTERT)
**HS737T**	Bone	0.89	Giant cell sarcoma
**HS821T**	Bone	0.89	Giant cell sarcoma
**HS822T**	Bone	0.89	Ewing’s sarcoma
**TE125T**	Soft_tissue	0.89	Rhabdomyosarcoma
**HLFA**	Lung	0.89	Epidermoid carcinoma
**HS618T**	Lung	0.89	Adenocarcinoma
**HS940T**	Skin	0.89	malignant melanoma
**HS742T**	Breast	0.88	Scirrhous adenocarcinoma
**B cell lines**		0.88	

## Discussion

HCC is one of the most lethal cancers worldwide, and advanced-stage patients or those with recurrent disease have poor prognosis owing to a lack of effective therapies. A wide gap remains between the demand for effective drugs for treating HCC and the successful development of such drugs [27]. Therefore, we reviewed the history of anticancer drug development for HCC. Since systemic therapy with cytotoxic agents was never approved for HCC, only molecular target drugs were evaluated. The FDA has not approved any molecular target drug for which the preclinical studies were performed using cell lines in cluster B. The approved drugs sorafenib [10,11], lenvatinib [28], regorafenib [29], and cabozantinib [30] were all developed using the cluster A cell lines HepG2, PLC_PRF_5, or Hep 3B2.1–7. This raises the possibility that the gene expression profiles of cluster B cell lines could be associated to a greater resistance to the drugs compared with that of cluster A cell lines. It is interesting to note that the cluster A cell lines were established from 1976 to 1990 and the cluster B cell lines were established from 1975 to 1999 (more than half of the cluster B cell lines were established after 1995). No patient related to the cluster A cell lines received preoperative anti-cancer drug treatment, but 6 out of 13 patients related to the cluster B cell lines received preoperative treatment (see Table 1). Therefore, it is very possible that some of the cluster B cell lines might be derived from treatment-resistant liver cancer cells. In this case, it would be beneficial to include cluster B cell lines as well as the cluster A cell lines for testing new effective treatments for HCC.

Transcriptomes enable us to compare the gene expression pattern between cancer cell lines and clinical human cancer tissues. For instance, Chen et al. [31] compared the gene expression profiles of 200 HCC samples from TCGA and over 1,000 cancer cell lines, including 25 liver cancer cell lines from the CCLE. They reported that the gene expression pattern of half of the liver cancer cell lines did not correlate with the gene expression profiles of clinical HCC samples. Intriguingly, the cancer cell lines determined to be dissimilar to HCC are consistent with those classified under cluster B in this study.

Recently, Caruso et al. [32] performed an unsupervised consensus classification of RNA sequencing data of 34 liver cancer cell lines and classified them into 3 subgroups: hepatocyte-like, mesenchymal cell-like and pluripotent cell-like groups. Of note, eight of the ten cell lines in our cluster A (Hep 3B2.1–7, Hep G2, huH1, HuH6, HuH7, JHH5, JHH7 and PLC_PRF_5) appeared in their hepatocyte-like group, suggesting that our classification of hepatocyte-like phenotype was similar to theirs. In order to overcome the limited coverage of previously studied liver cancer cell lines, Qiu et al. [33] built the Liver Cancer Model Repository (LIMORE) by establishing 50 new liver cancer cell lines derived from patients and collecting 31 commercially available liver cancer cell lines; and genomic and transcriptomic analyses for 81 liver cancer cell lines and related clinical samples brought on discerning understanding of liver cancer. These studies and our findings would provide valuable resources for liver cancer research.

It has been hypothesized that cancer cells may acquire fibroblast-like properties via epithelial to mesenchymal transition (EMT), which plays a role in cancer progression. However, it is difficult to recognize EMT processes in clinical practice, as EMT-related biomarkers are yet to be identified [34]. Moreover, it remains to be determined whether the gene expression pattern in experimentally induced EMT is similar to that in cluster B cell lines. In experiments involving EMT induced by gene transfer to HepG2 [35,36] or by TGF-β administration to HepG2 or Huh7 [37,38], the gene expression pattern of cells did not reveal similarity with that of cluster B, suggesting that the characteristics of cluster B cell lines are not acquired via EMT. It has been reported that the integration of HBV DNA into host cellular DNA altered cellular gene expression [39], and the expression of HBV proteins could have a direct effect on cellular functions and promote transformation [40]. Indeed, HBV induces EMT via HBx protein in vitro [41]. HBV has been also reported to be capable of infecting various cells, including stromal cells [42]. As shown in Table 1, five of the ten cell lines in cluster A, eight of the thirteen cell lines in cluster B, and one of the five cell lines in group C were HBV positive. These results indicated that integrated HBV DNA might not necessarily induce a fibroblast-like phenotype, and have a limited impact on the clustering analysis at least in our study.

Heterogeneous cell populations are observed in the tumor stroma of HCC and may contain fibroblasts named cancer-associated fibroblasts (CAFs). Our results did not provide evidence of the origin of the cluster B cell line, which was established from human HCC. However, the possibility that its origin was CAFs rather than liver cancer cells cannot be excluded.

In summary, liver cancer cell lines were classified into two main clusters by unsupervised clustering gene expression analysis. One cluster had a gene expression pattern similar to those of primary hepatocytes and most clinical HCC specimens. The other had a gene expression profile resembling that of the fibroblasts, and a few clinical HCC specimens exist with a similar profile. This demonstrates the usefulness of referring to datasets in public databases to select cell lines according to their gene expression profile and could potentially improve future studies in the field of cancer and anticancer drug development. The limitation of our study is that the expression patterns of cell lines are not completely consistent with those of clinical specimens. A possible cause of this discrepancy is that the bulk of tumor cells, including stromal cells, was analyzed for the clinical study. This is in contrast to the cell line analysis of almost monoclonal cells. Single-cell analysis, which has advanced considerably in recent years, can facilitate comparing the expression patterns of cell lines and clinical cancer cells.

This study, comparing the properties of various cell lines using the public database, provides a promising strategy for selecting the appropriate cell line.

## Supporting information

S1 Fig Overlaid scores and loadings plots. The liver cancer cell lines were clearly clustered into two groups. Clusters A (red plots) and B (blue plots) show negative and positive PC1 values, respectively. The loading plots of A genes (orange vector) and B genes (green vectors) also showed negative and positive PC1 values, respectively. Thus, these genes contributed to the separation of cluster A and B cell lines.
(TIF)

S1 Table Manifest for TCGA. (XLSX)

S2 Table Digestive cancer cell lines. (XLSX)

S3 Table Gene expression data of liver cancer cell lines. (XLSX)

S4 Table Gene expression data of hepatocellular carcinoma tumor samples. (XLSX)

S5 Table Gene expression data of liver cancer cell lines and primary cells. (XLSX)

S6 Table Cell lines from several organs. (XLSX)

S1 Document Calculation of representative vectors for each cell type. (DOCX)
