# Supplementary figures and images for: Gene expression profiles of liver cancer cell lines reveal two hepatocyte-like and fibroblast-like clusters
Source: PLoS One. 2021 Feb 4;16(2):e0245939. doi: 10.1371/journal.pone.0245939 (PMC7861371; doi:10.1371/journal.pone.0245939)

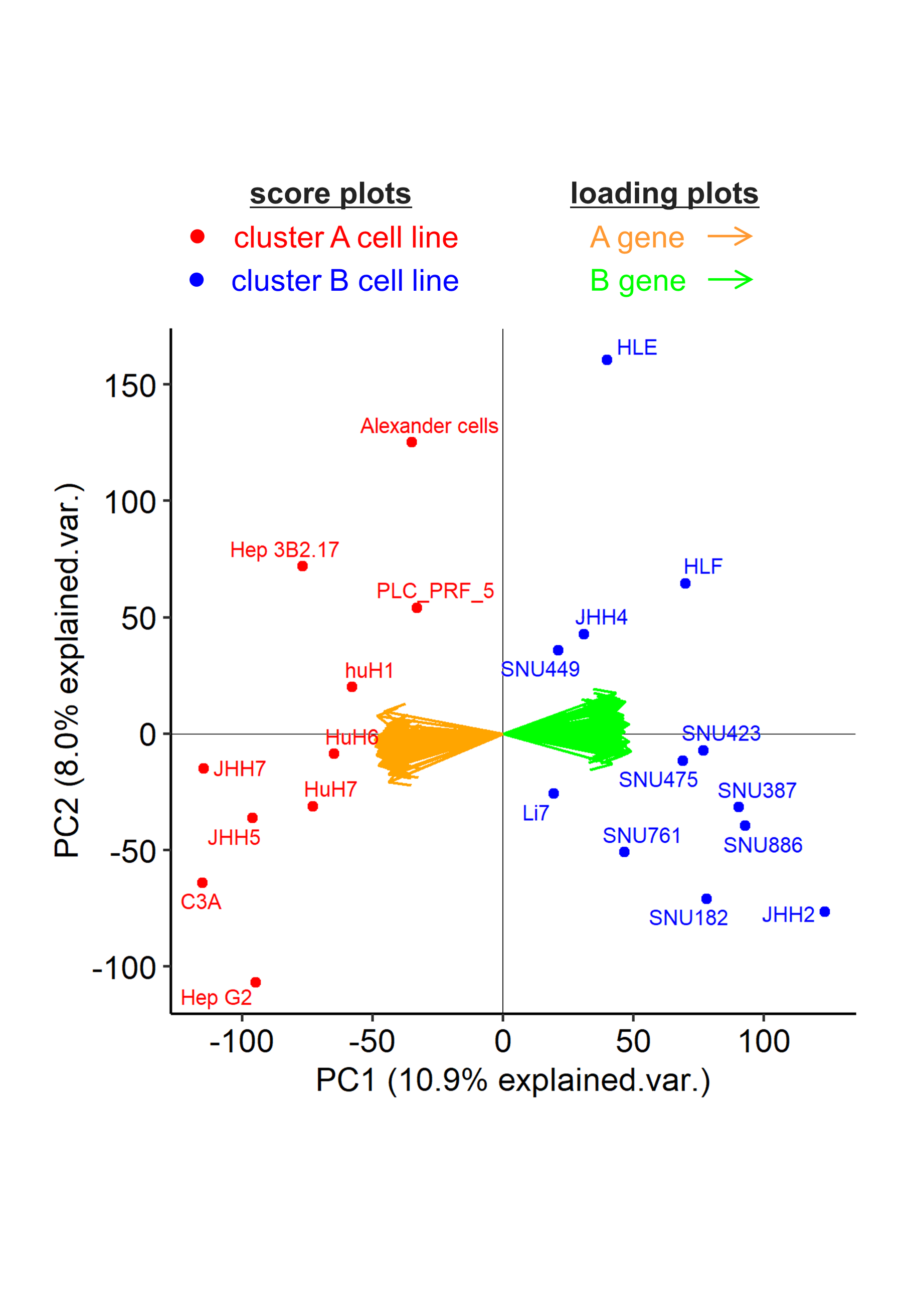

Supplement: S1 Fig — The liver cancer cell lines were clearly clustered into two groups. Clusters A (red plots) and B (blue plots) show negative and positive PC1 values, respectively. The loading plots of A genes (orange vector) and B genes (green vectors) also showed negative and positive PC1 values, respectively. Thus, these genes contributed to the separation of cluster A and B cell lines. (TIF) [file pone.0245939.s001.tif]
