# Supplementary material for: Gene expression profiles of liver cancer cell lines reveal two hepatocyte-like and fibroblast-like clusters
Source: PLoS One. 2021 Feb 4;16(2):e0245939. doi: 10.1371/journal.pone.0245939 (PMC7861371; doi:10.1371/journal.pone.0245939)
Supplement: S1 Document — (DOCX) [file pone.0245939.s008.docx]

**S1 Document**

**Calculation of representative vectors for each cell type**

The vector of the expression pattern for each cell type was generated as the log2 median of each probe set ID value as follows:

1. A given dataset contained limited cell types, and the number of cell types was presented as $L(L\mathbb{\in N})$. One cell type in the dataset was presented as $l \left( 1 \leq l \leq L \right)$*.*
2. Each cell type has several samples in the dataset. Multiple samples belonging to cell type $l$ were presented as $M_{l} \left( M_{l}\mathbb{\in N,}l=1,2,\ldots L, \sum_{1}^{L} M_{l}=L \right)$. One sample in cell type $l$ was presented as $m_{l} \left( 1 \leq m_{l} \leq M_{l} \right)$.
3. Each sample has the log2 converted signal intensity values of the fixed number of probe set IDs. The number of the whole probe set ID was $N \left( N\mathbb{\in N} \right)$ and that of one probe set ID is presented as $n \left( 1 \leq n \leq N \right)$.
4. One log2 converted signal intensity value of cell type $l$, sample $m_{l}$, and the probe set ID $n$ was presented as $x_{l, m_{l},n}$.
5. A representative value of cell type $l$ and probe set ID $n$ was obtained, as below.

$$y_{l,n}=median(x_{l, 1,n}, x_{l, 2,n},\ldots x_{l, M_{l},n})$$

6. A representative vector of cell type $l$ was obtained, as below.

$$z_{l}=(y_{l,1}, y_{l,2},\ldots y_{l,N})$$

7. Each $z_{l}$ was compared with each other.

Used packages

1 For clustering analysis among several cell lines.

Plot figure 1

gtable_0.2.0 [1]

gridExtra_2.2.1 [2]

hgu133plus2cdf_2.18.0 [3]

extrafontdb_1.0 [4]

extrafont_0.17 [5]

ggdendro_0.1-20 [6]

magrittr_1.5 [7]

ggplot2_2.2.1 [8]

GGally_1.3.2 [9]

stringr_1.2.0 [10]

R.utils_2.5.0 [11]

R.oo_1.21.0 [12]

R.methodsS3_1.7.1 [12]

MASS_7.3-47 [13]

affy_1.54.0 [14]

Biobase_2.36.2 [15]

BiocGenerics_0.22.0 [15]

BiocInstaller_1.26.0 [14]

2 For analysis of liver cancer cell lines and identifying DEGs.

Plot Figure 2a

scales_0.4.1 [16]

gridExtra_2.2.1 [2]

reshape2_1.4.2 [17]

hgu133plus2cdf_2.18.0 [3]

extrafontdb_1.0 [4]

extrafont_0.17 [5]

limma_3.32.5 [18]

ggdendro_0.1-20 [6]

ggplot2_2.2.1 [8]

magrittr_1.5 [7]

affy_1.54.0 [14]

Biobase_2.36.2 [15]

BiocGenerics_0.22.0 [15]

MASS_7.3-47 [13]

gplots_3.0.1 [19]

stringr_1.2.0 [10]

R.utils_2.5.0 [11]

R.oo_1.21.0 [12]

R.methodsS3_1.7.1 [12]

BiocInstaller_1.26.0 [20]

3 For analysis TCGA HCC samples in terms of DEGs.

Plot Figure 2b

forcats_0.2.0 [21]

dplyr_0.7.4 [22]

purrr_0.2.4 [23]

readr_1.1.1 [24]

tidyr_0.7.2 [25]

tibble_1.4.1 [26]

tidyverse_1.2.1 [27]

GenomicDataCommons_1.0.5 [28]

magrittr_1.5 [7]

scales_0.5.0 [16]

ggdendro_0.1-20 [6]

gridExtra_2.3 [2]

reshape2_1.4.3 [17]

ggplot2_2.2.1 [8]

DESeq2_1.16.1 [29]

SummarizedExperiment_1.6.5 [30]

DelayedArray_0.2.7 [31]

matrixStats_0.52.2 [32]

Biobase_2.36.2 [15]

GenomicRanges_1.28.6 [15]

GenomeInfoDb_1.12.3 [33]

IRanges_2.10.5 [34]

S4Vectors_0.14.7 [35]

BiocGenerics_0.22.1 [15]

extrafont_0.17 [5]

RTCGAToolbox_2.6.0 [36]

limma_3.32.10 [18]

gplots_3.0.1 [19]

MASS_7.3-49 [13]

stringr_1.2.0 [10]

BiocInstaller_1.26.1 [20]

4 For gene enrichment analysis via DAVID.

Plot Figure 3a

RDAVIDWebService_1.14.0 [37]

GOstats_2.42.0 [38]

Category_2.42.1 [39]

Matrix_1.2-10 [40]

AnnotationDbi_1.38.1 [41]

IRanges_2.10.2 [34]

S4Vectors_0.14.3 [35]

Biobase_2.36.2 [15]

graph_1.54.0 [34]

BiocGenerics_0.22.0 [15]

extrafont_0.17 [5]

clusterProfiler_3.4.4 [42]

DOSE_3.2.0 [43]

ggplot2_2.2.1 [8]

MASS_7.3-47 [13]

stringr_1.2 [10]

BiocInstaller_1.26.0 [20]

5 For comparison of DEGs between liver cancer cell lines and primary cells.

Plot Figure 3b

scales_0.4.1 [16]

ggdendro_0.1-20 [6]

gridExtra_2.2.1 [2]

reshape2_1.4.2 [17]

doParallel_1.0.10 [44]

foreach_1.4.3 [45]

affy_1.54.0 [14]

GEOquery_2.42.0 [46]

Biobase_2.36.2 [15]

BiocGenerics_0.22.0 [15]

RobLox_1.0 [47]

RobAStBase_1.0.1 [48]

rrcov_1.4-3 [49]

robustbase_0.92-7 [50]

distrMod_2.6.1 [51]

RandVar_1.0.1 [52]

distrEx_2.6.1 [53]

distr_2.6.2 [53]

SweaveListingUtils_0.7.7 [54]

sfsmisc_1.1-1 [55]

startupmsg_0.9.4 [56]

iterators_1.0.8 [57]

extrafont_0.17 [5]

ggplot2_2.2.1 [8]

MASS_7.3-47 [13]

stringr_1.2.0 [10]

BiocInstaller_1.26.0 [20]

7 For analysis and visualization of correlation analysis regarding CCLE expression array searching for immortalized fibroblasts.

Plot Figure 4

gtable_0.2.0 [1]

ggdendro_0.1-20 [6]

gridExtra_2.3 [2]

bindrcpp_0.2 [58]

doParallel_1.0.11 [44]

iterators_1.0.9 [57]

foreach_1.4.4 [45]

GEOquery_2.42.0 [46]

Biobase_2.36.2 [15]

BiocGenerics_0.22.1 [15]

extrafont_0.17 [5]

magrittr_1.5 [7]

dplyr_0.7.4 [22]

ggplot2_2.2.1 [8]

MASS_7.3-49 [13]

stringr_1.2.0 [10]

BiocInstaller_1.26.1 [20]

**References**

1. Wickham H. gtable: Arrange "Grobs" in Tables. 2016. Available from: https://CRAN.R-project.org/package=gtable
2. Auguie B. gridExtra: Miscellaneous Functions for "Grid" Graphics. 2016. Available from: https://CRAN.R-project.org/package=gridExtra
3. Project TB. hgu133plus2cdf: hgu133plus2cdf. 2015. Available from: <https://CRAN.R-project.org/package=hgu133plus2cdf>
4. Chang W. extrafontdb: Package for holding the database for the extrafont package. 2012. Available from: <https://CRAN.R-project.org/package=extrafontdb>
5. Chang W. extrafont: Tools for using fonts. 2014. Available from: https://CRAN.R-project.org/package=extrafont
6. Vries AD, Ripley BD. ggdendro: Create Dendrograms and Tree Diagrams Using "ggplot2". 2016. Available from: https://CRAN.R-project.org/package=ggdendro
7. Bache SM, Wickham H. magrittr: A Forward-Pipe Operator for R. 2014. Available from: https://CRAN.R-project.org/package=magrittr
8. Wickham H. ggplot2: Elegant Graphics for Data Analysis. New York: Springer; 2009. Available from: https://CRAN.R-project.org/package=ggplot2
9. Schloerke B. GGally: Extension to "ggplot2". 2017. Available from: https://CRAN.R-project.org/package=GGally
10. Wickham W. stringr: Simple, Consistent Wrappers for Common String Operations. 2017. Available from: https://CRAN.R-project.org/package=stringr
11. Bengtsson H. R.utils: Various Programming Utilities. 2016. Available from: https://CRAN.R-project.org/package=R.utils
12. Bengtsson H. The R.oo package - Object-Oriented Programming with References Using Standard R Code. In: Hornik K, Leisch F, Zeileis A, editors. Proceedings of the 3rd International Workshop on Distributed Statistical Computing (DSC 2003).
13. Venables WN, Ripley BD. Modern Applied Statistics with S. 4th ed. New York: Springer; 2002.
14. Gautier L, Cope L, Bolstad BM, Irizarry RA. affy--analysis of Affymetrix GeneChip data at the probe level. Bioinformatics. 2004;20(3): 307–315.
15. Amezquita RA, Lun A, Becht E, VCarey VJ, Carpp LN, Geistlinger L, et al. Orchestrating single-cell analysis with Bioconductor. Nat Methods. 2020;17(2): 137–145.
16. Wickham H. scales: Scale Functions for Visualization. 2016. Available from: https://CRAN.R-project.org/package=scales
17. Wickham H. Reshaping Data with the reshape Package. J Stat Softw. 2007;21(12): 1–20.
18. Matthew ER, Belinda P, Di W, Yifang H, Charity WL, Wei S, et al. limma powers differential expression analyses for RNA-sequencing and microarray studies, Nucleic Acids Res. 2015;43(7): 47.
19. Warnes GR. gplots: Various R Programming Tools for Plotting Data. 2016. Available from: https://CRAN.R-project.org/package=gplots
20. Tenenbaum D, Team B. BiocInstaller: Install/Update Bioconductor, CRAN, and github Packages. 2017. Available from: https://https://rdrr.io/bioc/BiocInstaller
21. Wickham H. forcats: Tools for Working with Categorical Variables (Factors). 2017. Available from: https://CRAN.R-project.org/package=forcats
22. Wickham H, Francois R, Henry L, Müller K. dplyr: A Grammar of Data Manipulation. 2017. Available from: https://CRAN.R-project.org/package=dplyr
23. Henry L, Wickham H. purrr: Functional Programming Tools. 2017. Available from: https://CRAN.R-project.org/package=purrr
24. Wickham H, Hester J, Francois R. readr: Read Rectangular Text Data. 2017. Available from: https://CRAN.R-project.org/package=readr
25. Wickham H, Henry L. tidyr: Easily Tidy Data with "spread()" and "gather()" Functions. 2017. Available from: https://CRAN.R-project.org/package=tidyr
26. Kirill M, Wickham H. tibble: Simple Data Frames. 2017. Available from: https://CRAN.R-project.org/package=tibble
27. Wickham H. tidyverse: Easily Install and Load "Tidyverse" Packages. 2017. Available from: https://CRAN.R-project.org/package=tidyverse
28. Morgan M, Sean D. GenomicDataCommons: NIH / NCI Genomic Data Commons Access. 2017. Available from: https://gdc.cancer.gov/content/genomicdatacommons-r-package
29. Love MI, Huber W, Anders S. Moderated estimation of fold change and dispersion for RNA-seq data with DESeq2. Genome Biology 2014:15(12): 550.
30. Morgan M, Obenchain V, Hester J, Pagès H. SummarizedExperiment: SummarizedExperiment container. 2017. Available from: https://bioconductor.org/packages/SummarizedExperiment
31. Pagès H. DelayedArray: Delayed operations on array-like objects. 2017. Available from: https://bioconductor.org/packages/DelayedArray
32. Bengtsson H. matrixStats: Functions that Apply to Rows and Columns of Matrices (and to Vectors). 2017. Available from: https://CRAN.R-project.org/package=matrixStats
33. Arora S, Morgan M, Carlson M, Pagès H. GenomeInfoDb: Utilities for manipulating chromosome and other "seqname" identifiers. 2017. Available from: https://bioconductor.org/packages/GenomeInfoDb
34. Lawrence M, Huber W, Pagès H, Aboyoun P, Carlson M, Gentleman R, et al. Software for Computing and Annotating Genomic Ranges. PLOS Computat Biol. 2013;9(8): e1003118.
35. Pagès H, Lawrence M, Aboyoun P. S4Vectors: S4 implementation of vectors and lists. 2017. Available from: https://bioconductor.org/packages/S4Vectors
36. Samur MK. RTCGAToolbox: a new tool for exporting TCGA Firehose data, PLOS ONE. 2014:9(9): e106397.
37. Fresno C, Fernández EA. RDAVIDWebService: a versatile R interface to DAVID, Bioinformatics. 2013;29(21): 2810–2811.
38. Falcon S, Gentleman R. Using GOstats to test gene lists for GO term association. Bioinformatics. 2007;23: 257–258.
39. Falcon RG, Sarkar D. Category: Category Analysis. 2017. Available from: https://bioconductor.org/packages/Category
40. Bates D, Maechler M. Matrix: Sparse and Dense Matrix Classes and Methods. 2017. Available from: https://CRAN.R-project.org/package=Matrix
41. Pagès H, Carlson M, Falcon S, Li N. AnnotationDbi: Annotation Database Interface. 2017. Available from: https://bioconductor.org/packages/AnnotationDbi
42. Yu G, Wang LG, Han Y, He QY. clusterProfiler: an R package for comparing biological themes among gene clusters. OMICS. 2012;16(5): 284–287.
43. Yu G, Wang LG, Yan GR, He QY. DOSE: an R/Bioconductor package for Disease Ontology Semantic and Enrichment analysis. Bioinformatics. 2015;31(4): 608–609.
44. R Analytics, Weston S. doParallel: Foreach Parallel Adaptor for the "parallel" Package. 2015. Available from: https://CRAN.R-project.org/package=doParallel
45. R Analytics, Weston S. foreach: Provides Foreach Looping Construct for R. 2015. Available from: https://CRAN.R-project.org/package=foreach
46. Davis S, Meltzer P. GEOquery: a bridge between the Gene Expression Omnibus (GEO) and BioConductor. Bioinformatics. 2007;23(14): 1846–1847.
47. Kohl M, Ruckdeschel P. RobLox: Optimally robust influence curves and estimators for location and scale. 2016. Available from: https://CRAN.R-project.org/package=RobLox
48. Kohl M, Ruckdeschel P. RobAStBase: Robust Asymptotic Statistics. 2017. Available from: https://CRAN.R-project.org/package=RobAStBase
49. Todorov V, Templ M, Filzmoser P. Detection of multivariate outliers in business survey data with incomplete information. Adv Data Anal Classif. 2011;5(1): 37–56.
50. Maechler M. robustbase: Basic Robust Statistics. 2016. Available from: https://CRAN.R-project.org/package=RobAStBase
51. Kohl M, Ruckdeschel P. R Package distrMod: S4 Classes and Methods for Probability Models. J Stat Softw. 2010;35(10): 1–27.
52. Kohl M, Ruckdeschel P. RandVar: Implementation of random variables. 2017.Available from: https://CRAN.R-project.org/package=RandVar
53. Ruckdeschel P, Kohl M, Stabla T, Camphausen F. S4 Classes for Distributions, R News. 2006;6(2): 2–6.
54. Ruckdeschel P. SweaveListingUtils: Utilities for Sweave Together with TeX "listings" Package. 2017. Available from: https://CRAN.R-project.org/package=SweaveListingUtils
55. Martin M. sfsmisc: Utilities from "Seminar fuer Statistik" ETH Zurich. 2017. Available from: https://CRAN.R-project.org/package=sfsmisc
56. Ruckdeschel P. startupmsg: Utilities for Start-Up Messages. 2017. Available from: https://CRAN.R-project.org/package=startupmsg
57. R Analytics, Weston S. iterators: Provides Iterator Construct for R. 2015. Available from: https://CRAN.R-project.org/package=iterators
